# Association between circulating full-length angiopoietin-like protein 8 and non-high-density lipoprotein cholesterol levels in Chinese non-diabetic individuals: a cross-sectional study

**DOI:** 10.1186/s12944-018-0802-9

**Published:** 2018-07-18

**Authors:** Song Yang, Xiaolu Jiao, Xiaoguang Huo, Miaomiao Zhu, Yi Wang, Xiangnan Fang, Yunyun Yang, Weidong Yue, Yanwen Qin

**Affiliations:** 1 Beijing An Zhen Hospital, Beijing Institute of Heart, Lung and Blood Vessel Diseases, Capital Medical University, No. 2 Anzhen Road, Chaoyang District, Beijing, 100029 China; 2 Zibo Central Hospital, Zibo, 255000 Shandong Province China; 3 Kailuan General Hospital Affiliated to North China University of Science and Technology, Tangshan, 063000 Hebei Province China; 4 The Second Affiliated Hospital of Harbin Medical University, Harbin, 150000 China

**Keywords:** Angiopoietin-like protein 8, Betatrophin, Dyslipidemia, Non-high-density lipoprotein cholesterol

## Abstract

**Background:**

Angiopoietin-like protein 8 (ANGPTL8) is a novel hormone involved in the regulation of lipid metabolism and glucose homeostasis. There are inconsistent results regarding the association between ANGPTL8 and lipids in humans. We aimed to investigate the associations between ANGPTL8 and lipids in people without diabetes.

**Methods:**

This was a cross-sectional study of 107 patients with dyslipidemia and 141 patients without. Dyslipidemia diagnosis was based on Chinese guidelines for the prevention and treatment of dyslipidemia in adults. Total cholesterol (TC), triglycerides (TG), low-density lipoprotein cholesterol, and high-density lipoprotein cholesterol (HDL-C) were examined. Non-HDL-C was calculated by subtracting HDL-C from TC. Circulating full-length ANGPTL8 concentrations were measured using enzyme-linked immunosorbent assay. Associations between log-transformed circulating full-length ANGPTL8 and serum lipids were examined using multivariate linear regression analysis.

**Results:**

Circulating ANGPTL8 concentrations were significantly elevated in patients with dyslipidemia compared with patients without dyslipidemia. Circulating full-length ANGPTL8 concentrations were positively associated with non-HDL-C, TG and TC levels after adjusting for age, gender, body mass index, high-sensitivity C-reactive protein, alanine aminotransferase, and creatinine.

**Conclusion:**

In people without diabetes, circulating full-length ANGPTL8 concentrations in patients with dyslipidemia were significantly elevated compared with non-dyslipidemia, and ANGPTL8 was positively associated with serum non-HDL-C levels.

**Electronic supplementary material:**

The online version of this article (10.1186/s12944-018-0802-9) contains supplementary material, which is available to authorized users.

## Background

Dyslipidemia includes a broad spectrum of lipid abnormalities [1] and is an independent risk factor for atherosclerotic cardiovascular disease (CVD) [2–4].The prevalence of dyslipidemia is 40.4% among adults in China [5]. Evidence based on results from multiple randomized controlled trials that revealed that CVD could be prevented by reducing and low-density lipoprotein cholesterol (LDL-C) is strong and compelling [1]. However, recent guidelines suggest that non-high-density lipoprotein cholesterol (HDL-C) is a stronger independent risk factor for CVD and should be considered as a risk marker and a secondary treatment target for CVD [4, 5]. Besides, patients with dyslipidemia are also at high risk of insulin resistance [6], fatty liver [7] and other diseases. Serum lipid profiles are affected by various factors, such as postprandial state and age. Recently, angiopoietin-like protein 8 (ANGPTL8) was identified as a novel hormone that plays a significant role in lipid metabolism [8–10].

ANGPTL8, also known as betatrophin [11], TD26 [12], re-feeding induced fat and liver [13], lipasin [14] and PRO1185 [15], is an atypical member of the ANGPTL family [9]. It is expressed in both adipose tissue and liver in mice but only in liver in humans [8]. ANGPTL8 is reported to be a secreted protein involved in the regulation of lipid [15–17] and glucose metabolism [18, 19].

Based on meta-analyses of a non-obese population, circulating ANGPTL8 concentrations in patients with type 2 diabetes mellitus are elevated compared with non-diabetic adults [20]. Overexpression of ANGPTL8 in mouse livers did not alter beta cell expansion or glucose metabolism but increased serum lipid levels [21, 22]. However, there are inconsistent results regarding the relationship between ANGPTL8 and serum lipids in human studies. Studies have reported that ANGPTL8 concentrations are significantly and positively associated with triglycerides (TG) and LDL-C levels but inversely associated with HDL-C levels in patients with diabetes [23, 24]. In contrast, it has been reported that ANGPTL8 concentrations were not associated with lipid profiles [25], while Javier Gómez-Ambrosi et al. reported that circulating ANGPTL8 concentrations were significantly lower in people with dyslipidemia characterized by low HDL-C or high TG levels [26]. The discrepancy in the findings may be partly because of hyperglycemia, which is associated with circulating ANGPTL8 concentrations [11, 27–29].

In the current study, we investigated circulating full-length ANGPTL8 concentrations in patients with dyslipidemia and the association between full-length ANGPTL8 concentrations and serum lipids in Chinese people without diabetes.

## Methods

### Participants

The study was designed as cross-sectional study and was conducted between March 2016 and April 2017 at the Arteriosclerosis Clinic at Beijing An Zhen Hospital. A total of 1601 consecutive patients were eligible for the study. Patients with diabetes, abnormal glucose tolerance, secondary hyperlipidemia, pregnancy, cancer, acute infectious diseases, hepatic dysfunction, or abnormal renal function were excluded. Patients taking lipid-lowering drugs were also excluded. A final population of 248 participants was enrolled in the study. According to diagnostic standards, participants were divided into two groups: dyslipidemia (*n* = 107) and non-dyslipidemia (*n* = 141). The dyslipidemia diagnosis was based on Chinese guidelines on prevention and treatment of dyslipidemia in adults [5]. At least one of the following was present in patients with dyslipidemia: high TG, high TC, high LDL-C, high non-HDL-C, and low HDL-C levels. In contrast, the non-dyslipidemia group displayed normal lipid levels. High serum TG level was defined as serum TG ≥1.7 mmol/L. High serum TC level was defined as serum TC ≥5.2 mmol/L. High serum LDL-C level was defined as serum LDL-C ≥ 3.4 mmol/L. High serum non-HDL-C level was defined as serum non-HDL-C ≥ 4.1 mmol/L. Low serum HDL-C level was defined as serum HDL-C < 1.0 mmol/L [5]. Finally, 107 patients with dyslipidemia were included in the study, this comprised 16 patients with hypertriglyceridemia, four with hypercholesterolemia, 24 with low serum HDL-C only, and 63 with combined hyperlipidemia. Diagnoses of diabetes and abnormal glucose tolerance were based on the criteria of the American Diabetes Association [30]. According to the recommendations of the Health Promotion Administration, overweight was defined as BMI ≥24 kg/m^2^, and normal weight as a 24 > BMI ≥ 18.5 kg/m^2^ [31]. All participants provided written informed consent before enrollment. The study was approved by the Medicine Ethics Committee of Beijing An Zhen Hospital (#2017010X) and was conducted in accordance with the principles contained within the Declaration of Helsinki.

### Anthropometric measurements

Anthropometric determinations and blood extractions were performed on a single day. Height and weight were measured with participants wearing light indoor clothing and being barefoot using calibrated portable electronic weighing scales and portable inflexible height measuring bars. Blood pressure was measured after a 5-min rest in the sitting position. Electronic sphygmomanometer was used to measure the blood pressure***.*** Blood pressure was determined at least three times using the right upper arm, and the mean value was used in the analyses. BMI was calculated using the standard BMI formula: body mass (in kilograms) divided by square of height (in meters).

### Blood sample preparation

All blood samples were collected after participants had fasted overnight. Clinical variables included TG, TC, LDL-C, HDL-C, fasting blood glucose, Hs-CRP, alanine aminotransferase, aspartate aminotransferase, γ-glutamyltransferase, uric acid, and creatinine. Serum TG, TC, LDL-C, and HDL-C levels and other routine serum biochemical parameters were measured using a biochemical analyzer (Hitachi-7600; Hitachi, Tokyo, Japan). Serum non-HDL-C was calculated by subtracting HDL-C from TC according to the 2016 ESC/EAS Guidelines for the Management of Dyslipidemias [4]. All measurements were obtained using blinded quality control specimens at the Department of the Biochemical Laboratory at Beijing An Zhen Hospital. Blood samples were centrifuged for 10 min at 3000 rpm and 4 °C. Plasma samples were subsequently stored in a freezer at − 80 °C. Circulating full-length ANGPTL8 concentrations were measured using an enzyme-linked immunosorbent assay (ELISA) kit (cat.No.11644 h; Wuhan ELAAB Science, Wuhan, China) according to the manufacturer’s instructions, with the intra- and inter-assay coefficients of variation less than 5 and 10%, respectively. The detection limit for the ELISA assay was 78–5000 pg/mL.

### Statistical analysis

Continuous variables are expressed as mean ± standard deviations or median (interquartile range) and categorical variables as numeral (percentage). Independent Student’s *t*-tests for normal distribution and Wilcoxon rank sum tests for asymmetric distribution were used to analyze the differences in continuous variables. Chi-squared tests and Fisher’s exact tests were used to analyze categorical variables. The associations between ANGPTL8 and serum lipids were determined using multivariate linear regression analyses. Log transformation was used for variables that were asymmetrically distributed. A *P*-value < 0.05 was considered statistically significant. Statistical analyses were performed using SPSS 20.0 (IBM Corp, Armonk, NY).

## Results

### Baseline clinical characteristics of the study population

This study included 107 patients with dyslipidemia and 141 participants without. The clinical characteristics of the study population were shown in Table 1. There were no differences in age (*P* = 0.983), gender (*P* = 0.510), or fasting blood glucose levels (*P* = 0.744) between the two groups. Compared with the non-dyslipidemia group, the dyslipidemia group had significantly higher BMI (25.47 ± 2.95 vs. 24.52 ± 3.05 kg/m^2^, *P* = 0.014), but there were no differences in the numbers of overweight people (*P* = 0.596) or postmenopausal women (*P* = 0.551) between the two groups. Patients with dyslipidemia also had higher high-sensitivity C-reactive protein (Hs-CRP) levels (*P* < 0.001). Remarkably, circulating full-length ANGPTL8 concentrations were significantly elevated in the dyslipidemia group compared with the non-dyslipidemia group (506.18 [368.31–723.56] vs. 408.21 [294.52–511.41] pg/mL, *P* < 0.001) (Table 1).

**Table 1:** Anthropometric and biochemical characteristics of the subjects included in the study

	Dyslipidemia *n* = 107	Non-dyslipidemia *N* = 141	P
Age (years)	51.70 ± 11.64	51.74 ± 14.86	0.983
Male (n,%)	69 (64.49%)	84 (59.57%)	0.510
BMI (kg/m^2^)	25.47 ± 2.95	24.52 ± 3.05	0.014*
Overweight (n,%)	70 (65.42%)	87 (61.70%)	0.596
Postmenopausal women (n,%)	21 (55.26%)	31(54.39%)	0.551
SBP (mmHg)	126.54 ± 17.43	120.52 ± 17.51	0.005*
DBP (mmHg)	78.67 ± 11.33	74.94 ± 13.46	0.002*
FBG (mmol/L)	5.17 ± 0.42	5.16 ± 0.52	0.744
TG (mmol/L)	1.82 (1.26–2.29)	0.93 (0.68–1.19)	< 0.001**
TC (mmol/L)	4.60 ± 1.08	4.13 ± 0.56	< 0.001**
LDL-C (mmol/L)	2.86 ± 0.94	2.37 ± 0.48	< 0.001**
HDL-C (mmol/L)	1.00 ± 0.24	1.33 ± 0.23	< 0.001**
Non-HDL-C (mmol/L)	3.60 ± 0.96	2.81 ± 0.51	< 0.001**
UA (umol/L)	338.52 ± 81.00	318.09 ± 76.88	0.044*
CR (umol/L)	71.98 ± 14.61	71.22 ± 14.17	0.683
ALT (U/L)	24.18 ± 18.55	18.82 ± 9.13	0.001*
AST (U/L)	22.50 ± 11.13	20.91 ± 5.23	0.652
γ-GT (U/L)	28.26 ± 16.88	25.62 ± 16.10	0.078
Hs-CRP (mg/L)	1.31 (0.46–2.80)	0.60 (0.33–1.86)	< 0.001**
ANGPTL8 (pg/ml)	506.18 (368.31–723.56)	408.21 (294.52–511.41)	< 0.001**

Results are expressed as mean ± standard deviation, median (interquartile range) or n (%). Differences between groups were analyzed by independent Student t-test, Fisher’s exact test, χ^2^ text, or Wilcoxon test. Abbreviations: BMI body mass index, SBP systolic blood pressure, DBP diastolic blood pressure, FPG fasting plasma glucose, TG triglycerides, TC total cholesterol, LDL-C low-density lipoprotein cholesterol, HDL-C high-density lipoprotein cholesterol, UA uric acid, CR creatinine, AST aspartate aminotransferase, ALT alanine aminotransferase, γ-GT γ-glutamyltransferase, Hs-CRP high-sensitivity C-reactive protein, ANGPTL8 angiopoietin-like protein 8

*P < 0.05, **P < 0.001

### Linear regression analyses of relationships between ANGPTL8 and serum lipids

Associations between log-transformed circulating full-length ANGPTL8 and serum lipids were examined using multivariate linear regression analyses. As shown in Table 2, circulating ANGPTL8 concentrations were positively associated with non-HDL-C (β = 0.042, 95% confidence interval (CI): 0.010–0.074, *P* = 0.011) and TG (β = 0.279, 95%CI: 0.172–0.386, *P* < 0.001) levels but negatively associated with HDL-C levels (β = − 0.105, 95%CI: − 0.199 – − 0.011, *P* = 0.029). No associations with LDL-C (β = 0.025, 95%CI: − 0.011 – 0.061, *P* = 0.176) or TC (β = 0.028, 95%CI: − 0.003 – 0.060, *P* = 0.081) levels were observed. After adjusting for age, gender, BMI, alanine aminotransferase, Hs-CRP, and creatinine, circulating ANGPTL8 concentrations were positively associated with non-HDL-C (β = 0.050, 95%CI: 0.020–0.079, P < 0.001), TG (β = 0.271, 95%CI: 0.166–0.376, P < 0.001), and TC (β = 0.039, 95%CI: 0.009–0.068, *P* = 0.009) levels and negatively associated with HDL-C levels (β = − 0.108, 95%CI: − 0.201 – − 0.014, *P* = 0.024). There was no association between circulating ANGPTL8 and serum LDL-C (β = 0.031, 95%CI: − 0.002 – 0.064, *P* = 0.064).

**Table 2:** Multivariate linear regression analysis of log-transformed ANGPTL8 and clinical variables

	Unadjusted Β 95%CI	*P*-Value	Model 1 Β 95% CI	*P*-Value	Model 2 Β 95% CI	*P*-Value
Non-HDL-C	0.042 (0.010–0.074)	0.011*	0.053 (0.023–0.083)	< 0.001**	0.050 (0.020–0.079)	< 0.001**
T1	Reference		Reference		Reference	
T2	0.116 (0.025–0.207)	0.013*	0.112 (0.027–0.198)	0.010*	0.107 (0.025–0.189)	0.011*
T3	0.108 (0.023–0.194)	0.014*	0.239 (0.086–0.392)	0.002*	0.208 (0.060–0.357)	0.006*
Log-TG	0.279 (0.172–0.386)	< 0.001**	0.265 (0.156–0.375)	< 0.001**	0.271 (0.166–0.376)	< 0.001**
TC	0.028 (− 0.003–0.060)	0.081	0.044 (0.013–0.074)	0.005*	0.039 (0.009–0.068)	0.009*
HDL-C	−0.105 (− 0.199 - -0.011)	0.029*	− 0.104 (− 0.199 - -0.008)	0.033*	−0.108 (− 0.201 - -0.014)	0.024*
LDL-C	0.025 (−0.011–0.061)	0.176	0.042 (0.008–0.076)	0.016*	0.031 (− 0.002–0.064)	0.064

Tertile values of non-HDL-C are expressed as T1 (≤4.1 mmol/L), T2 (4.1–4.9 mmol/L), T3 (> 4.9 mmol/L)

Dependent variable: log-transformed ANGPTL8

Model 1: adjusted for age, gender and BMI

Model 2: adjusted for Model 1 + ALT, Hs-CRP,CR

Abbreviations: *ANGPTL8* angiopoietin-like protein 8, *CI* confidence interval, *HDL-C* high-density lipoprotein cholesterol, *TG* triglycerides, *TC* total cholesterol, *LDL-C* low-density lipoprotein cholesterol, *BMI* body mass index, *ALT* alanine aminotransferase, *Hs-CRP* High-sensitivity C-reactive protein, *CR* creatinine

*P < 0.05, **P < 0.001

## Discussion

In this study, we found that circulating full-length ANGPTL8 concentrations in patients with dyslipidemia were significantly elevated compared with those without dyslipidemia. Circulating full-length ANGPTL8 concentrations were positively associated with serum non-HDL-C, TG, and TC levels but negatively associated with HDL-C levels after adjusting for confounding factors.

ANGPTL8 plays an important role in lipid metabolism [13, 32]. Compared with wild-type littermates, *Angptl8-*knockout mice displayed slower weight gain and decreased plasma TG levels but increased lipoprotein lipase (LPL) activity [9]. Adenoviral ANGPTL8 overexpression in mice increased serum TG levels, while recombinant ANGPTL8 inhibited LPL activity [8]. This could be the mechanism by which ANGPTL8 is linked to the regulation of serum lipids. LPL hydrolyzes TG from chylomicrons and very LDL to form free fatty acids, which are taken up by peripheral tissues, including fat, muscle, and heart [33].

ANGPTL8 is a new but atypical member of the ANGPTL family, because it lacks the C-terminal fibrinogen-like domain but shares a common coiled-coil domain at the N-terminus with ANGPTL3 and ANGPTL4 [33]. The N-terminal coiled-coil domain is involved in lipid regulation [34] by directly inhibiting LPL activity [35]. ANGPTL8 may activate another ANGPTL family member, ANGPTL3. It was reported that ANGPTL8 coimmunoprecipitated with the N-terminal domain of ANGPTL3 in mouse plasma and increased the appearance of N-terminal ANGPTL3 in cultured hepatocytes [35]. ANGPTL3 activation results in very low LDL-C, HDL-C, and TG levels. It is reported that ANGPTL3 deficiency reduces the risk of coronary heart disease in humans [36]. A human monoclonal antibody against ANGPTL3, named evinacumab, which was derived using Regeneron’s Velocimmune® technology platform and is a fully human monoclonal antibody with high affinity to ANGPTL3 from mouse, rat, monkey, and humans [37]. It is reported that evinacumab caused a dose-dependent placebo-adjusted reduction in fasting TG levels of up to 76% and LDL-C levels of up to 23% in humans, and resulted in a greater decrease in atherosclerotic lesion area and necrotic content compared with a control antibody in mouse model of dyslipidemia [37]. The impact of ANGPTLs on plasma lipid levels has resulted in increased scrutiny of ANGPTLs as therapeutic targets for dyslipidemia and atherosclerotic cardiovascular disease [17].

In the current study, we found that circulating full-length ANGPTL8 concentrations positively correlated with serum non-HDL-C levels. Non-HDL-C, which represents the total cholesterol content of apolipoprotein B containing lipoproteins [38], includes the TG-rich lipoproteins (very LDL, intermediate-density lipoproteins, and remnants) [39]. Non-HDL-C is strong predictive factor that directly reflects the total number of atherogenic lipoprotein particles [40]. Non-HDL-C is calculated by subtracting HDL-C from TC and can be obtained in the non-fasting state without affecting results [38]. Recent guidelines suggested that non-HDL-C is a stronger independent risk factor for CVD and should be considered as a risk marker and a secondary treatment target for CVD [4]. Some researchers have even proposed that non-HDL-C is a better predictor of coronary atherosclerosis severity than LDL-C [41, 42].

We found that there was no association between circulating full-length ANGPTL8 levels and serum LDL-C. To the best of our knowledge, ANGPTL8 play an important role in TG metabolism by inhibiting LPL activity instead of cholesterol metabolism [16, 35]. It is reported that ANGPTL8 gene mutation results in HDL-C levels were increased by 10 mg/dl, and TG levels were decreased by 15% but LDL-C levels were not changed [43].

We also found that patients with dyslipidemia in the current study exhibited a dramatic increase in circulating full-length ANGPTL8 concentrations, circulating full-length ANGPTL8 concentrations were positively associated with serum TG and TC levels and negatively associated with HDL-C levels. We also found that circulating ANGPTL8 concentrations positively correlated with age (Additional file 1: Table S1). Epidemiological evidence indicates that blood lipid levels increase with increased age [44], which could be the reason for the elevation in ANGPTL8 concentrations.

We found no association between circulating ANGPTL8 concentrations and BMI in the current study (Additional file 1: Table S1), consistent with the results of Roth et al. [45]. However, to the best of the authors’ knowledge, results regarding the association between ANGPTL8 concentrations and BMI are inconsistent. Some researchers have indicated that ANGPTL8 concentrations tended to negatively correlate with BMI [29] while others observed that plasma ANGPTL8 concentrations positively correlated with BMI [29]. It is possible that the inconsistency may be associated with the origins of obesity. It is reported that circulating ANGPTL8 concentrations are different in people with obesity depending on the origins of obesity [46] which may be linked to body composition, basal metabolic rate, or metabolic outcomes [47]. To avoid a confounding effect, we adjusted for age and BMI in this study. Circulating ANGPTL8 concentrations are elevated in patients with non-alcoholic fatty liver disease [48] and liver steatosis [46]. It is also reported that there is a positive association between circulating ANGPTL8 and serum Hs-CRP [11]. Renal function is independently associated with circulating ANGPTL8 and circulating ANGPTL8 concentrations positively correlate with serum creatinine [49]. To avoid a confounding effect, we also adjusted for alanine aminotransferase, Hs-CRP, and creatinine in this study.

It is reported that ANGPTL8 requires ANGPTL3 for its effects on LPL [50], therefore, we also measured the circulating ANGPTL3 concentration. There was no difference in circulating ANGPTL3 concentrations between the dyslipidemia and no-dyslipidemia groups (*P* = 0.097) (Additional file 2: Figure S1), consistent with the study by Guo et al. [51], nor was there any association between circulating ANGPTL3 and ANGPTL8 concentrations (*P* = 0.160 and *P* = 0.100, respectively) (Additional file 1: Table S1). Co-expression of ANGPTL3 and ANGPTL8 in mice resulted in a reduction in circulating ANGPTL3 and an increase in plasma TG levels, whereas plasma TG levels did not change with expression of ANGPTL8 alone [35]. Inhibition of ANGPTL8 in mice using a monoclonal antibody decreased plasma TG levels and increased LPL activity [50]. It is therefore suggested that ANGPTL8 plays a role in serum lipid metabolism either directly or indirectly (by promoting the cleavage of serum ANGPTL3).

The enzyme-linked immunosorbent assay (ELISA) kits used in the current study recognize the N-terminus of ANGPTL8, and ANGPTL8 is likely cleaved in vivo to release C-terminal fragments. The N-terminal kit measures the full-length protein while the C-terminal kit measures total ANGPTL8 species, including both the full-length protein and C-terminal fragments [29]. ANGPTL8 concentrations determined using the N-terminal kit were lower than that determined using the C-terminal kit, resulting in low values (in pg/mL) for ANGPTL8 being obtained in the current study.

This study had some limitations. First, it was a cross-sectional study, which means it can only demonstrate associations, not causality. Second, the study had a small sample size and larger samples are needed to confirm the results. Third, ANGPTL8 may be associated with inflammation [52]. Inflammatory cytokines, such as interleukin-6, were not measured. Whether inflammation played a role in the altered plasma ANGPTL8 concentrations could not be determined. Finally, as all study participants were Chinese, the findings may not be generalizable to other ethnicities. The findings should be confirmed in other populations.

## Conclusion

In conclusion, we found that non-diabetic people with dyslipidemia had significantly increased full-length ANGPTL8 concentrations compared with those without dyslipidemia. Circulating full-length ANGPTL8 concentrations were positive associated with serum non-HDL-C levels.

## Additional files

Additional file 1 **Table S1.** Spearman correlations between ANGPTL8 and clinical variables. (DOCX 24 kb)

Additional file 2 **Figure S1.** The levels of circulating ANGPTL3 in patients with dyslipidemia and no-dyslipidemia. (JPG 70 kb)

## Abbreviations

ANGPTL8: Angiopoietin-like protein 8
BMI: Body mass index
CVD: Cardiovascular disease
HDL-C: High-density lipoprotein cholesterol
Hs-CRP: High-sensitivity C-reactive protein
LDL-C: Low-density lipoprotein cholesterol
LPL: Lipoprotein lipase
TC: Total cholesterol
TG: Triglycerides
