# Supplementary material for: Association between circulating full-length angiopoietin-like protein 8 and non-high-density lipoprotein cholesterol levels in Chinese non-diabetic individuals: a cross-sectional study
Source: Lipids Health Dis. 2018 Jul 18;17:161. doi: 10.1186/s12944-018-0802-9 (PMC6052512; doi:10.1186/s12944-018-0802-9)
Supplement: Supplementary file 1 — Table S1. Spearman correlations between ANGPTL8 and clinical variables. (DOCX 24 kb) [file 12944_2018_802_MOESM1_ESM.docx]

Table S1: Spearman correlations between ANGPTL8 and clinical variables.

|  | All subjects | |
| --- | --- | --- |
|  | ρ | P-value |
| Age (years) | 0.333 | <0.001** |
| BMI (kg/m^2^) | -0.015 | 0.814 |
| SBP (mmHg) | 0.114 | 0.072 |
| DBP (mmHg) | 0.074 | 0.244 |
| FBG (mmol/L) | 0.171 | 0.007* |
| TG (mmol/L) | 0.285 | <0.001** |
| TC (mmol/L) | 0.067 | 0.297 |
| LDL-C (mmol/L) | 0.016 | 0.805 |
| HDL-C (mmol/L) | -0.133 | 0.036* |
| No-HDL-C (mmol/L) | 0.087 | 0.173 |
| UA (umol/L) | 0.289 | <0.001** |
| CR (umol/L) | 0.290 | <0.001** |
| ALT (U/L) | 0.033 | 0.569 |
| AST (U/L) | 0.105 | 0.069 |
| γ-GT (U/L) | 0.591 | <0.001** |
| Hs-CRP (mg/L) | 0.117 | 0.043* |
| ANGPTL3 (ng/ml) | 0.160 | 0.100 |

* P<0.05, ** P<0.001.

Table 2: Anthropometric and biochemical characteristics of all the subjects included in the study.

| Age (years) | 51.72±13.54 |
| --- | --- |
| Male (n,%) | 153(61.7%) |
| BMI (kg/m^2^) | 24.93±3.04 |
| Overweight (n,%) | 157(63.3%) |
| Postmenopausal women (n,%) | 52(54.7%) |
| SBP (mmHg) | 123.36±17.51 |
| DBP (mmHg) | 76.31±12.04 |
| FBG (mmol/L) | 5.17±0.48 |
| UA (umol/L) | 326.90±79.18 |
| CR (umol/L) | 71.55±14.34 |
| ALT (U/L) | 21.13±14.21 |
| AST (U/L) | 21.60±8.23 |
| γ-GT (U/L) | 26.75±16.45 |
| Hs-CRP (mg/L) | 1.63±2.72 |
