# Supplementary figures and images for: Association between circulating full-length angiopoietin-like protein 8 and non-high-density lipoprotein cholesterol levels in Chinese non-diabetic individuals: a cross-sectional study
Source: Lipids Health Dis. 2018 Jul 18;17:161. doi: 10.1186/s12944-018-0802-9 (PMC6052512; doi:10.1186/s12944-018-0802-9)

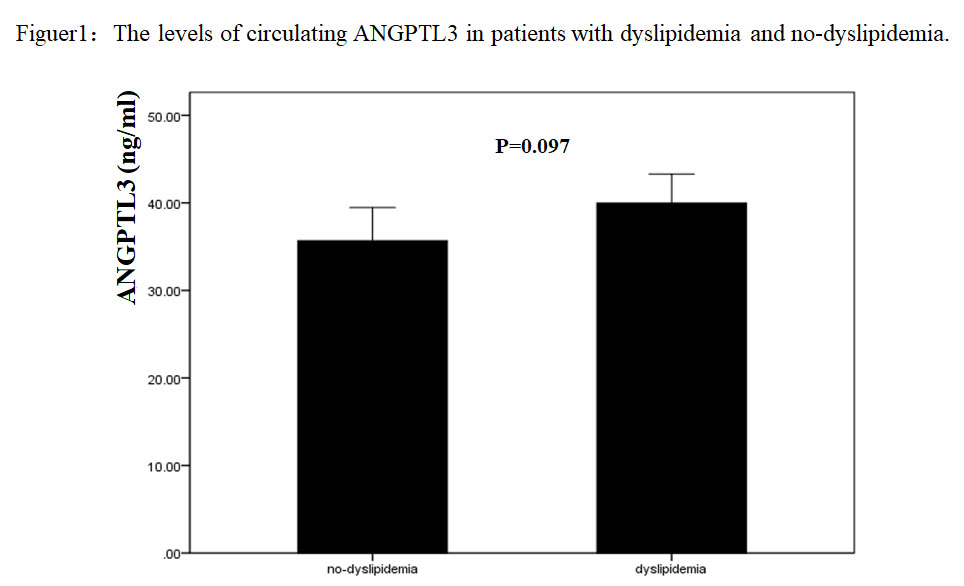

Supplement: Supplementary file 2 — Figure S1. The levels of circulating ANGPTL3 in patients with dyslipidemia and no-dyslipidemia. (JPG 70 kb) [file 12944_2018_802_MOESM2_ESM.jpg]
